# Medical Opinion and Movement

**Published:** 1908-10-03

**Authors:** 


### Medical Opinion and Movement.

THE jubilee of the Emperor Francis Joseph of Austria has moved the community of Vienna to build another large hospital in the capital. Vienna, as is well known, is not very well provided with hospitals, and there are far more cases than the existing institutions, large as they are, can accommodate. The new hospital is to be called the Jubilee Hospital, and a sum of ten million kronen (about £40,000) has been voted by the City Council as a preliminary instalment for its construction. It will be built on the outskirts of the city, and will consist of a general hospital of 850 beds, a series of private wards accommodating 130 patients, and a convalescent institute. The private wards will be arranged on the sanatorium system, and will be equipped according to the most modern principles, while the hospital itself will be divided into the usual divisions, of which the medical will contain 300 beds, and the surgical 130. In addition there will be gynæcological, ophthalmological, and dermatological wards, and a special division for tuberculous patients. As this hospital will only be able to cope with the patients from one quarter of the city, it has been decided to build a separate hospital for another district. The plans for this latter institution, however, have not yet been drawn up. When these new clinics are completed Vienna will be in a much better position to deal with the vast amount of clinical material which at the present time leads to such overcrowding in its old hospitals.

THE pathology of hæmophilia is so little known that there can at present be no question of dealing with this disease in a radical manner. Empirical treatment, which is mainly symptomatic, has been tried since the condition was first described, and hardly a year passes without some new revelation regarding the possibilities of this or that method of treatment. Unfortunately all these lauded remedies and preventives, from calcium salts to injections of senna decoction, have proved hopeless failures. The latest addition to this host of empirical measures which the practitioner can use in treating a case of hæmophilia is injection of serum. First tried by the French and Italian practitioners in the early part of 1900, it has since then been used by many physicians, and some supporters of this method have expressed themselves so enthusiastically that one would believe that the certain cure for hæmophilia has at last been discovered. By injections of antidiphtheritic serum it is possible, according to these enthusiasts, so to enhance the coagulability of the hæmophilic's blood that the surgeon need not fear to undertake an operation on such a patient. If antidiphtheritic serum fails any other variety of serum may be used.

A NUMBER of observations have been made, and results are published from time to time to prove the success of this new method. Thus Lommel of Jena, records the latest case in which he made use of the method. The patient, "a hæmophilic boy aged four years," suffered from violent epistaxis during an attack of whooping cough. As all other measures proved unavailing to stop the bleeding, Lommel injected antistreptococcic serum twice, on the first occasion 20 c.c., and on the second 10 c.c., and plugged the child's nostrils with pellets of gauze soaked in the serum. The bleeding stopped, and the child made a good recovery. In all such cases the question of diagnosis should be more fully discussed than it usually is, since it is impossible to accept every case of violent epistaxis as being due to hæmophilia. The statement that a patient suffers from hæmophilia should be supported by some definite evidence, when new lines of treatment for hæmophilia are recommended. Serum injections, however, seem to be based on a reasonable principle in such cases, and are worth a trial where other measures fail.

LOWY, of Karlsbad, does not agree with the statement of a well-known physician who declared that "an acute attack of coryza lasts three weeks, and when the physician treats it, twenty-one days." On the contrary, he urges an active treatment of every case of coryza. He has found great benefit from a 10 per cent. solution of protargol, which is sprayed into the anterior nares, or applied by means of a fine camel's hair brush to all parts of the nasal mucosa. This remedy suffices for all early cases, one or two applications of the solution being usually sufficient. When the case is advanced, the brushing must be repeated several times a day or tampons soaked in the protargol solution must be inserted into the nostrils. He has used the solution with success in old cases of rhinitis, and has found it answer excellently in the majority of cases. Where the solution is not well tolerated he employs a mixture of camphor and menthol (four parts menthol and one part camphor). A few drops of this heavy oily liquid are added to a small amount of water in a test tube which is heated over a spirit lamp. The patient holds the tube under his nostrils, and draws the vapour into the anterior nares, taking care that the liquid does not spurt. Another mixture he employs is oleum menth. pip. in alcohol and water, equal parts, which is similarly inhaled. Internally he gives his patients 15 grains of aspirin. Above all he urges them to inspire and expire with the mouth wholly closed, and in all cases he lays stress on the importance of nasal respiration in the treatment of chronic cases.

GUERSANT has reported an interesting case of empyema treated by Bier's method. For lesions of the extremities the hyperæmic method is, of course, well known, and is now coming into general use, but few practitioners are as yet aware of the value of this method in inflammatory conditions of internal organs. On the Continent, however, it is extensively employed in thoracic and even abdominal conditions such as nephritis. M. Guersant's case was that of a child with a large right-sided empyema, which had been discharging for four months, and had not proved amenable to the ordinary treatment. Bier's treatment, by suction cups applied over the sinus, was systematically tried with excellent results, the child being discharged a month after the treatment was commenced. The suction cups were applied several times a day, and no attempt was made to drain the cavity between the sittings. Under the treatment the general health rapidly improved. The method is applicable to many thoracic lesions, and in the discussion which followed the reading of M. Guersant's paper before the Societé Centrale de Médicine, M. Pencot alluded to a case of intrathoracic abscess which he had successfully treated in the same manner. Special suction cups are necessary with carefully modelled edges, so as to fit close to the concavity of the chest wall. The treatment must be graduated with considerable care at first, as it may otherwise give rise to constitutional disturbance, especially in debilitated children.

---

MANGEAIS, in a recent thesis presented to the University of Paris, attempts a discussion of one of the most interesting questions in renal surgery, namely, the influence of which a diseased kidney exerts upon the sound organ on the other side. Most surgeons are familiar with the fact that in cases of tubercular kidney disease, where the lesion is in the majority of cases unilateral, there are often traces of albumen in the urine excreted by the kidney on the other side, although that organ can scarcely be regarded as diseased. Similarly in unilateral pyonephrosis of calculous origin, catheter specimens of urine from the ureter on the sound side also show traces of albumen. In cases where there is no question of suppuration such abnormalities are not found in the excretion from the healthy organ. These facts have led Mangeais to study the matter experimentally, and the results of his work are certainly interesting, though his findings can hardly be called conclusive, and much confirmatory evidence is desirable before his theories can be accepted in their entirety by the medical profession.

---

IN nephrolithiasis he shows that one of the main factors is the amount of uric acid in the circulation. This throws an additional strain on the sound kidney when the tubules of one organ are diseased or when the functional activity of one kidney as a whole is lowered by the presence of obstructing calculi. In renal tuberculosis a similar strain is thrown on the healthy organ, which results in a unilateral polyuria, and, if the condition persists, in albuminuria and changes in the epithelium lining the glomeruli. In such cases no tuberculous change can be demonstrated in the healthy kidney; all that exists is a condition of wear and tear caused by overwork. A much more malign influence, however, is exerted when the diseased kidney, by throwing vicious products into the circulation, directly infects the sound kidney. Such cases are found in advanced tubercle, in septic conditions, and in carcinoma, in all of which the sound kidney may be infected from the diseased organ through the blood stream. Clinically, these cases are of far greater importance, as it is difficult to recognise them at the commencement, the changes in the sound kidney being apt to be put down to overwork strain.

---

CASES of tubercular prostate treated by removal of the gland are rare enough to make the record of the latest of some general interest. This was the case of a man aged 45 years, under the care of Dr. Pauchet, of Algiers, who was submitted to three operations before prostatectomy was finally performed. Eight years before the final operation double castration was performed for "general tuberculosis of the testicles," after which the patient, who when first seen was in a very asthenic condition, made a favourable recovery and was able to continue his work. Gradually, however, he began to complain of vesical and perineal pains, and was seen by several medical men who confirmed the diagnosis of prostatic tubercle. Eight years after the first operation a large swelling presented in the perineal region, and the patient had retention of urine. Perineal section was performed, and a foul abscess evacuated. The bladder was washed out, and a smaller perineal abscess opened. The patient was found to have a large vesico-rectal fistula, and the prostate was riddled with sinuses. The general health of the man was very bad, and there was grave doubt as to the condition of the kidneys. Nevertheless, Dr. Pauchet enucleated the prostate successfully by the perineal route, an operation which was attended with considerable difficulty. No sutures were inserted, the wound being left widely opened and gauze tampons being inserted. The patient made a slow recovery, and some months after the operation reported himself in fairly good health, although the perineal wound had not yet closed. The report of the case is unfortunately without an account of the pathological condition of the parts removed, but the condition of the patient was so bad that the success of the operation, in spite of its difficulties, must be considered as brilliant.

---

IN the *Berlinner Klinische Wochenschrifte*, Dr. Knopf has recently called attention to the utility of respiratory gymnastics in the treament of chronic bronchitis. According to the author, no bronchitic patient ever respires in a normal manner, and as a rule the lower portion of the thorax in such patients remains immobile. It is necessary that a thorough examination should be made of each patient to determine in what respects his respiratory movement differs from the normal, and to gauge this the patient must be stripped to the waist and examined in front of a mirror. In this manner deviations from the normal can easily be detected. When suitable exercises have been carried out the muscles of the abdominal wall quickly gain strength. The author points out that at the commencement of treatment the patient sometimes experiences a feeling of vertigo. In addition, pains in the abdominal and pectoral muscles are sometimes felt. These symptoms are transitory. It is well that the patient should be taught to cough only when there is some bronchitic secretion to expectorate. This habit is more easily acquired than would at first sight appear possible. The author concludes his paper by remarking that, with the exception of locomotion, respiration is the function which most depends on will power, and that it is consequently strange that up to now so little stress has been laid upon respiratory gymnastics by the medical faculty, while at the same time expectorants and soothing drugs are freely ordered despite the toxic effects which so frequently follow their administration.

---

Mr. T. G. Ouston, of Newcastle, has devised a new operation for depressed fractures of the nose, details of which were given by him at the last meeting of the British Medical Association. As pointed out by the author, fractures of the nasal septum and dislocations of the lateral cartilage not infrequently occur from blows and falls on the nose, especially in young children; and permanent external and internal deformities may result. Lateral displacements, giving rise to internal obstruction may be rectified, or at least improved, by present methods of operative procedure, but depressions of the nasal crest are less easy to treat with any success. The method devised by Mr. Ouston consists briefly in a separation of the nasal cartilages from the nasal bones and nasal processes of the maxillæ by means of a tenotome introduced subcutaneously through the side of the nose at the site of the depression. The separation is carried out so that the nasal structures corresponding to the depressed part can be lifted by the tenotome to the normal level. The nasal bones are then transfixed by a large strong needle as near their common anterior suture as possible. The depressed anterior portion is transfixed in a similar manner by a second needle, and the second needle is drawn upwards and forwards towards the second by means of strips of gauze carried round in a figure of eight fashion from one needle to the other on each side. The sides of the nose are protected from pressure by the gauze by means of small discs on the needles, adjustable with a screw "thread." The needles are kept in position for about ten days, by which time the parts should be fixed.

---

The researches of Overton and others have shown that the narcotic effect of anæsthetics is connected with their solvent action upon fats. Owing to this property they are rapidly taken by the red corpuscles of the blood, of which the stroma is rich in lecithin, and transferred to the system, where they rapidly diffuse by reason of the lipoids contained in the tissues. The nerve-tissues being especially rich in fats absorb a large proportion; hence the narcosis. None of the tissues, however, escape altogether the deleterious action of these drugs, owing to their ability to take up and fix them; and in consequence they all undergo certain changes after the narcosis. Such changes are evidenced by increased excretion of nitrogen and ammonia in the urine, glycosuria, and also sometimes albuminuria, and examination of the blood reveals lipæmia and acetonæmia. Dr. Karl Reicher has conducted some experiments on dogs to ascertain these various modifications in metabolism following anæsthesia caused by chloroform, ether, morphia, scopolamine-morphine, and alcohol respectively. Increased excretion of nitrogen was shown in all cases for two or three days following the anæsthesia, but it was especially marked with chloroform and alcohol and slight with ether. The excretion of ammonia amounted in all cases to double or triple the normal. Similarly the total amount of acetone eliminated was at least twice the normal quantity. Alcohol-ether extracts of the blood showed an increase of fatty substances, amounting to three or four times the normal in dogs. According to these researches the toxic effect of these drugs is approximately the same in all cases, and consists chiefly in a perversion of the normal metabolism of the system.

---

"Recurrent rheumatoid fever of menstruation"—such is the title proposed by Dr. M. G. Riebold for a morbid condition which has been the subject of attention of several observers in recent years, and to which this author has devoted special study. The condition is found chiefly among virgins, and consists in premenstrual attacks of fever accompanied by rheumatic manifestations of a more or less intense character, but without any definite signs of local inflammation. The temperature rises gradually and attains its maximum in the course of a few days. The duration of the fever does not exceed a week, and the temperature falls by lysis. During that time the patient presents a typhoid condition; she is apathetic, complains of headache, pains in the back and limbs, with anorexia, constipation, and furred tongue. In the course of the disease rheumatic symptoms appear, articular pains, and occasionally swelling of the joints. Cardiac symptoms, more or less severe, may occur; a rapid and feeble pulse, cardiac arrhythmia, systolic murmurs, cyanosis, dyspnœa, and other circulatory disturbances may be present. Occasionally traces of albumin may be detected in the urine. Such is the clinical picture drawn by the author, and it will be interesting to ascertain whether such a condition is confirmed by other observers as a separate morbid entity. Dr. Riebold connects the condition in some way with the function of menstruation, but is unable to throw further light upon its ætiology. He has not found any drug to have a specific influence upon the course of the disease. Salicylates have the effect of lowering the temperature, but do not affect the other symptoms nor shorten the duration of the attack. On the other hand, they appear to aggravate the circulatory troubles, and he has therefore discarded their use.

